# The Effect of Spatial Smoothing on Representational Similarity in a Simple Motor Paradigm

**DOI:** 10.3389/fneur.2017.00222

**Published:** 2017-05-29

**Authors:** Michelle H. A. Hendriks, Nicky Daniels, Felipe Pegado, Hans P. Op de Beeck

**Affiliations:** ^1^Laboratory of Biological Psychology, Brain and Cognition, KU Leuven, Leuven, Belgium

**Keywords:** functional magnetic resonance imaging, multi-voxel pattern analyses, spatial smoothing, primary motor cortex, auditory cortex

## Abstract

Multi-voxel pattern analyses (MVPA) are often performed on unsmoothed data, which is very different from the general practice of large smoothing extents in standard voxel-based analyses. In this report, we studied the effect of smoothing on MVPA results in a motor paradigm. Subjects pressed four buttons with two different fingers of the two hands in response to auditory commands. Overall, independent of the degree of smoothing, correlational MVPA showed distinctive patterns for the different hands in all studied regions of interest (motor cortex, prefrontal cortex, and auditory cortices). With regard to the effect of smoothing, our findings suggest that results from correlational MVPA show a minor sensitivity to smoothing. Moderate amounts of smoothing (in this case, 1−4 times the voxel size) improved MVPA correlations, from a slight improvement to large improvements depending on the region involved. None of the regions showed signs of a detrimental effect of moderate levels of smoothing. Even higher amounts of smoothing sometimes had a positive effect, most clearly in low-level auditory cortex. We conclude that smoothing seems to have a minor positive effect on MVPA results, thus researchers should be mindful about the choices they make regarding the level of smoothing.

## Introduction

Spatial smoothing is part of the preprocessing of functional magnetic resonance imaging (fMRI) data and is most often performed using a three-dimensional Gaussian filter (“kernel”) of several millimeters to filter the image. In this way, high-frequency information is removed, while low-frequency information remains (1). Smoothing is incorporated in the analysis of neuroimaging data for several reasons.

A first and extensive argument is a matter of spatial resolution inherent to the method of fMRI. Blood oxygen level dependent (BOLD) fMRI localizes changes in oxygenation levels in the blood that are related to synaptic activation (2). Hence, BOLD fMRI indirectly measures brain activity through a hemodynamic signal that is also spatially smoothed. The smallest unit of measurement used in fMRI, a voxel, still contains a myriad of neurons. In addition, the vascular response measured with fMRI expands over various millimeters, partially resulting from “draining veins” that remove oxygen-rich blood from the active voxels (3). This causes the point spread function (PSF) of the neural response to be spatially widened, which in turn results in weaker precision and thus a smaller resolution (4). The signal strength and resolution also seem to depend on voxel size, and there is a complex relation between voxel size and the width of the BOLD PSF (5). Furthermore, the spatial specificity depends upon the field strength of the magnet (2). This first argument shows the biggest limitations of fMRI as a method. Smoothing is therefore used to increase the signal-to-noise ratio for the larger-scale information that is relevant in most fMRI studies (1).

A second reason to smooth is related to its beneficial effect for the validity of statistical assumptions as incorporated in the well-known Random Field Theory (6). Finally, the fact that spatial smoothing helps to overcome the inter-individual differences in anatomy is another reason to smooth (6). Although the choice of a smoothing-level appears to be arbitrary, it might not be without consequence. It is one of several parameters that are often set “by default,” but might nevertheless influence the outcome of statistical analyses (1).

Despite the abundant use of spatial smoothing in fMRI research in general, it is much less commonly used in one specific type of fMRI analysis, namely, multi-voxel pattern analysis or MVPA. MVPA focuses upon patterns of activity across voxels instead of single-voxel activations (7). There are many types of MVPA, but our focus will be on the type used in this report: correlational MVPA. Correlational MVPA entails a split of data in two subsets, followed by a correlation between the neural activity pattern for conditions in one subset with the pattern of conditions in the other subset (8, 9), which has since been referred to as representational similarity analysis (10). An important approach to find out whether the neural patterns contain any information about the different conditions is to compare the correlation between same conditions with the correlation between different conditions. If the correlation between same conditions is reliably higher, this means that the patterns of brain activation provide reliable information about which condition was presented (7).

Op de Beeck (9) studied the sensitivity of MVPA results under different levels of smoothing in visual cortex. The motive for this study was the suggestion of so-called hyperacuity. Several authors suggested that MVPA allows to pick up brain maps that are organized at a scale that is finer than the voxel size, such as picking up the signals from orientation columns at sub-millimeter scale through voxels of 3 mm isotropic (11, 12). The authors did not perform spatial smoothing on their data and indeed report findings that suggest hyperacuity. However, Op de Beeck (9) showed that the outcome of MVPA is surprisingly robust to the level of spatial smoothing performed during preprocessing. More specifically, highly smoothed data contained at least a similar amount of information as data that were not smoothed. When using correlational MVPA, the correlations even increased with a larger amount of smoothing. Hence, MVPA indeed did not seem to pick up small-scale activation patterns. Since then, an intense debate has unfolded about the degree to which MVPA results are driven by small- and large-scale selectivity maps [in favor of small-scale or multiple-scale selectivity maps (13–17). In favor of large-scale selectivity maps (18–20)].

More in detail, Chaimow et al. (5) investigated the plausibility of several suggested mechanisms to account for the findings that support hyperacuity. A first hypothesized mechanism involves local, irregular, and arbitrary deviations in the functional organization of the brain at the columnar level, which can cause biases at the level of voxels, which can be picked up when performing MVPA (12, 21, 22). Each voxel contains columns with different orientation preferences. Importantly, a bias arises because these preferences are distributed unevenly across voxels. First, the authors found evidence for a contribution of low frequency as well as high-frequency components underlying these random variations. Second, draining veins are expected to play a role (12, 22), as voxels can include signals from larger blood vessels, causing a bias in decoding. Kriegeskorte et al. (23) proposed the third mechanism. Third, in their model, fMRI voxels are believed to act like spatiotemporal filters of neural activity, as every voxel samples from a unique structure of blood vessels. Fourth, Boynton (24) considers the fact that a voxel’s responses are biased toward certain orientations to be evidence that decoding can capture small-scale activations in the brain even though the resolution of fMRI is limited. This “aliasing” was found to be practically impossible for fMRI by Chaimow et al. (5), because features inherent to fMRI (width of the BOLD PSF and voxel size) behave like a low-pass filter, filtering out the small-scale information that this mechanism appears to rely upon. Finally, in contradiction with the previous mechanism, large-scale activations are suggested to play a role as well (18–20), for example, resulting from relationships between retinotopic location and preferences for particular orientations. For full explanations of the aforementioned mechanisms, we refer to the article of Ref. (5). Finally, Sengupta et al. (25) studied the effect of the acquisition resolution in V1 on the decoding of orientation. They suggest the opposing views are not mutually exclusive, namely, the idea that fMRI data “is broadband in nature” and contains both small-scale and large-scale activation patterns.

Investigation of the effect of smoothing can also provide information about the spatial organization of neural representations. To give just one example, Brants et al. (26) used a manipulation of spatial smoothing to study the spatial organization of ventral occipitotemporal cortex. They used correlational MVPA and found that correlations were higher when data were smoothed compared to non-smoothed data. Additionally, they examined whether this effect of smoothing was the same for all spatial scales, in this case contrasting conditions that are differentiated at the subordinate level (baby-face and elderly face, small spatial scale) or different categories (faces and houses, large spatial scale). They found an interaction effect in which the correlation increased less for the subordinate distinctions, suggesting that the selectivity maps related to these subordinate distinctions are organized at a finer spatial scale.

Up to now, these studies focused exclusively upon visual cortex. Here we explored the effect of smoothing on MVPA results in other brain regions in a paradigm that did not involve visual stimulation. In particular, we studied this effect in motor cortex, prefrontal cortex, low-level auditory cortex (A1), and high-level auditory cortex (A2). For this purpose, a fairly basic motor paradigm with auditory instructions was used. The first goal encompassed a study of the sensitivity of MVPA results to the level of smoothing. For this end, we used seven levels of smoothing (ranging from no smoothing to 15 mm FWHM in steps of 2.5 mm) and compared the results. A second goal was to investigate the extent to which smoothing would affect results with different spatial scales. For this reason, we included conditions that are expected to activate nearby parts of motor cortex, such as different fingers of the same hand, as well as conditions that should activate far-away parts of motor cortex in different hemispheres, such as fingers of a different hand. Based on previous findings, we predicted that a larger amount of smoothing would increase the difference in correlations between same and different conditions, predominantly for the largest spatial scale.

## Materials and Methods

### Participants

Data were acquired using eight healthy subjects (six female, mean age of 22.75 years, with a standard deviation of 3.06, one left-handed). All participants reported absence of neurological or psychiatric history. This study was carried out in accordance with the recommendations of the medical ethics committee of the KU Leuven with written informed consent from all subjects. All subjects gave written informed consent in accordance with the Declaration of Helsinki. The study served as a control experiment in a more extensive study, of which the protocol was approved by the medical ethics committee of the KU Leuven.

### Stimuli and fMRI Task

Eight auditory stimuli were recorded to serve as instructions. The words “left middle finger,” “left index finger,” “right index finger,” and “right middle finger” were used in the so-called Finger runs, and “one,” “two,” “three,” and “four” were used in the so-called Number runs. Stimuli were recorded by two voices (one male, one female) and were in Dutch.

**Figure 1:**
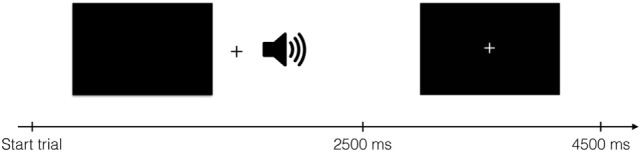
**Structure of one trial**. During auditory stimulus presentation, a black screen was shown. The auditory stimulus started 600 ms after the black screen appeared. After 2,500 ms, a fixation cross was shown for 2,000 ms. Each trial lasted for 4,500 ms in total.

Brain imaging data were collected while participants received these spoken instructions to press one of four buttons. Buttons were pressed using middle- and index fingers of both hands. There were thus four conditions, one for each finger. One run lasted for 504 s and consisted of 112 events of 4,500 ms, 96 of which were experimental events with stimulus presentation. Visually, each trial started with a blank screen presented for 2,500 ms, followed by a screen with a fixation spot for 2,000 ms (Figure 1). Although visual stimulation was not strictly necessary to complete the task, black screens and fixation crosses were used to ensure equal visual input for all participants throughout the experiment. The auditory cues were presented during the blank screen period. The experiment comprised four runs per participant; two “Finger”- and two “Number”-runs in randomized order. In each run, 202 volumes were acquired, starting approximately 5 s before stimulus/fixation presentation and ending approximately 5 s after stimulus/fixation presentation ended.

### fMRI Data Acquisition

Data were acquired using a 3 T Philips Ingenia CX scanner (Department of Radiology of KU Leuven). A 32-channel head coil was used. Functional data consisted of T2*-weighted echoplanar images (EPIs) with voxel size 2.52 mm × 2.58 mm × 2.5 mm, an interslice gap of 0.2 mm on top of the slice thickness of 2.5 mm, repetition time 2,550 ms, echo time 30 ms, acquisition matrix 84 × 82 voxels, 45 slices per volume acquired in ascending order, and a field of view (FOV) of 211 mm × 211 mm × 121 mm. Additionally, a high-resolution T1-weighted anatomical scan was acquired with voxel size of 0.98 mm × 0.98 mm × 1.2 mm, repetition time 9.6 ms, echo time 4.6 ms, acquisition matrix 256 × 256 voxels, and 182 slices. Stimuli were presented using Psychtoolbox 3 (27). Visual stimuli were projected on a screen using an NEC projector with a NP21LP lamp. The participant viewed the screen through a mirror attached to the head coil. Viewing distance was approximately 64 cm. Auditory stimuli were presented *via* headphones. Before starting, participants were asked to indicate whether the volume was of an acceptable level, to make sure they could easily understand the instructions.

### fMRI Data Analysis

Data were analyzed using two different approaches. A first approach follows the most frequently used pipeline of fMRI data analysis, in which smoothing is performed as a step during preprocessing. In contrast, smoothing will be performed after defining the general linear model (GLM) for the second approach, on anatomically masked beta-images.

#### Approach 1: Smoothing during Preprocessing

##### Preprocessing

Data were processed using the Statistical Parametric Mapping software package (SPM8, Wellcome Department of Cognitive Neurology, London, UK) and custom Matlab code (Mathworks, Inc.). Preprocessing involved correction of the functional images for slice timing differences and realignment with the mean image to correct for head motion. Functional and anatomical images were then co-registered, using the mean realigned image as reference image and normalized to the MNI template. In a final step of the first approach, normalized functional images were smoothed using Gaussian kernels with different full-width at half maxima (FWHM). The FWHM is related to standard deviation [2.55 times the standard deviation (1)]. In line with the goal of this writing, the data were analyzed using seven levels of smoothing ranging from no smoothing to a kernel of 15 mm × 15 mm × 15 mm (approximately six times the original voxel size), in steps of 2.5 mm (approximately the voxel size). The preprocessed data were used in further analyses.

##### General Linear Model

We modeled the onset of each trial of a condition by an event with a duration of 400 ms centered around each subjects’ finger movement to capture the signal in the motor cortex. This choice was made *a priori*, without further detailed analyses. The finger movement was operationalized by the reaction time of each individual subject measured for every trial in the scanner. The signal in each voxel was modeled for every subject using a GLM. This model was generated for each run and contained four regressors of interest (one for each stimulus condition, being four fingers) and six additional regressors to account for head motion (realignment parameters obtained during motion correction). The design matrix was created for each participant and contained four runs. After fitting the GLM, the parameter estimates were used to calculate each voxel’s response in each of the four conditions. This resulted in so-called “beta” values, which were used to perform MVPA.

##### Definition of Regions of Interest

We examined whether movement of different fingers can be distinguished based on neural activation patterns, for which we primarily focused on motor cortex. In addition, we also included prefrontal cortex and low- and high-level auditory cortex. Voxels were first selected based on a whole-brain univariate contrast of all four conditions minus baseline. We used a threshold of *p* < 0.001 (uncorrected for multiple comparisons). Voxels that were significantly activated by this contrast were selected for each participant. This selection was further restricted anatomically, by selecting those voxels that were conjointly present in the previous selection and anatomical masks of all studied regions. The anatomical masks were created with the anatomical WFU PickAtlas Toolbox (Wake Forrest University PickAtlas, http://fmri.wfubmc.edu/cms/software). Motor cortex was defined by Brodmann areas (BA) 4 (primary motor cortex) and 6 (premotor cortex) and contained 8,120 voxels. Prefrontal cortex was specified by the frontal lobe minus BA 4 and 6, which included 65,021 voxels. The low-level auditory cortex included BA 41 and 42 and comprised 762 voxels. High-level auditory cortex was defined by BA 22 and consisted of 1,820 voxels.

##### Correlational MVPA

**Figure 2:**
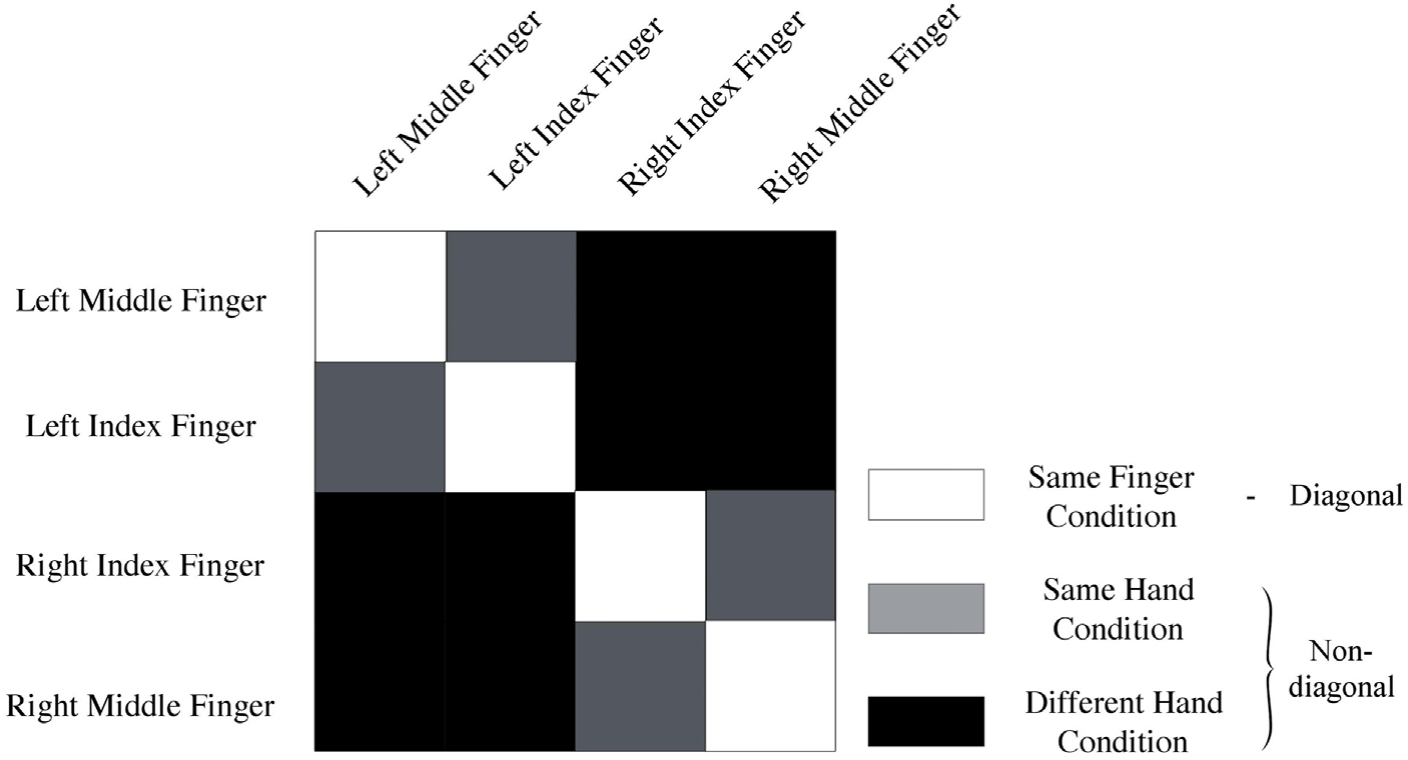
**Representation of conditions**. Same-fingers condition (white) entails correlations between same fingers. The same-hand condition (gray) entails correlations between different fingers on the same hand. Finally, the different-hand condition (black) entails correlations between fingers on different hands. In addition, the model used to analyze the data can be derived from this image. Diagonal (white) entails a correlation of neural activity for movement of the same fingers. On the non-diagonal (gray + black) cells contain a correlation between neural activation for movement of different fingers.

Data of every voxel within a region of interest were normalized for every run by cocktail blank subtraction of the mean response across all conditions (7, 28). The data were then split into two halves by randomly assigning the runs into two groups, and correlations between patterns of same versus different categories of stimuli were computed. This yielded a 4 × 4 correlation matrix. We computed the mean diagonal and non-diagonal of this matrix for every subject. Additionally, the difference between the averaged diagonal and non-diagonal was computed (Figure 2). When this difference is significant across subjects, we can infer that the multi-voxel patterns in a brain region contain information to distinguish between different conditions (i.e., different fingers). We will look how distinct levels of smoothing affect this difference between diagonal and non-diagonal.

##### Spatial Organization

Finally, we studied different expected scales of organization. Spatial scale was operationalized by comparing MVPA results of different conditions (Figure 2). More specifically, we looked at the mean correlation between same fingers (same-finger condition), same hand but different fingers (same-hand condition), and fingers on different hands (different-hand condition). We expected to find that the correlation between same fingers would be higher than the correlation between different fingers on the same hand, which in turn would be higher than the correlation between different fingers on different hands. This was operationalized by computing pairwise differences in mean correlations and studying their significance. We also studied whether there was an interaction between spatial scale and smoothing.

#### Approach 2: Smoothing after Masking ROIs

##### Preprocessing

Except for the smoothing in approach 1, data were processed in the same way. In approach 2, there was no smoothing during preprocessing.

##### General Linear Model

We modeled the GLM in the same way as above, with one exception the input data. Here the input data were the unsmoothed preprocessed data.

##### Anatomical Definition of Regions of Interest

Anatomical ROI were defined identically to the procedure for the first approach and used for the following step.

##### Masking of Beta-Images

This step is unique to the second approach. We used anatomical masks to isolate the information within the regions of interest and ignore the information outside these regions by treating these values as missing values (13).

##### Smoothing

Smoothing was performed on the masked beta-images. Equal to the first approach, data were smoothed using Gaussian kernels with FWHM ranging from no smoothing to a kernel of 15 mm × 15 mm× 15 mm in steps of 2.5 mm.

##### Definition of Regions of Interest

Regions of interest were defined identically to the procedure for the first approach.

##### Correlational MVPA

The multivariate analysis was performed in the same way as for the first approach.

##### Spatial Organization

The different spatial scales were examined in the exact same manner as for the first approach.

## Results

### Approach 1: Smoothing during Preprocessing

#### Effect of Smoothing

**Figure 3:**
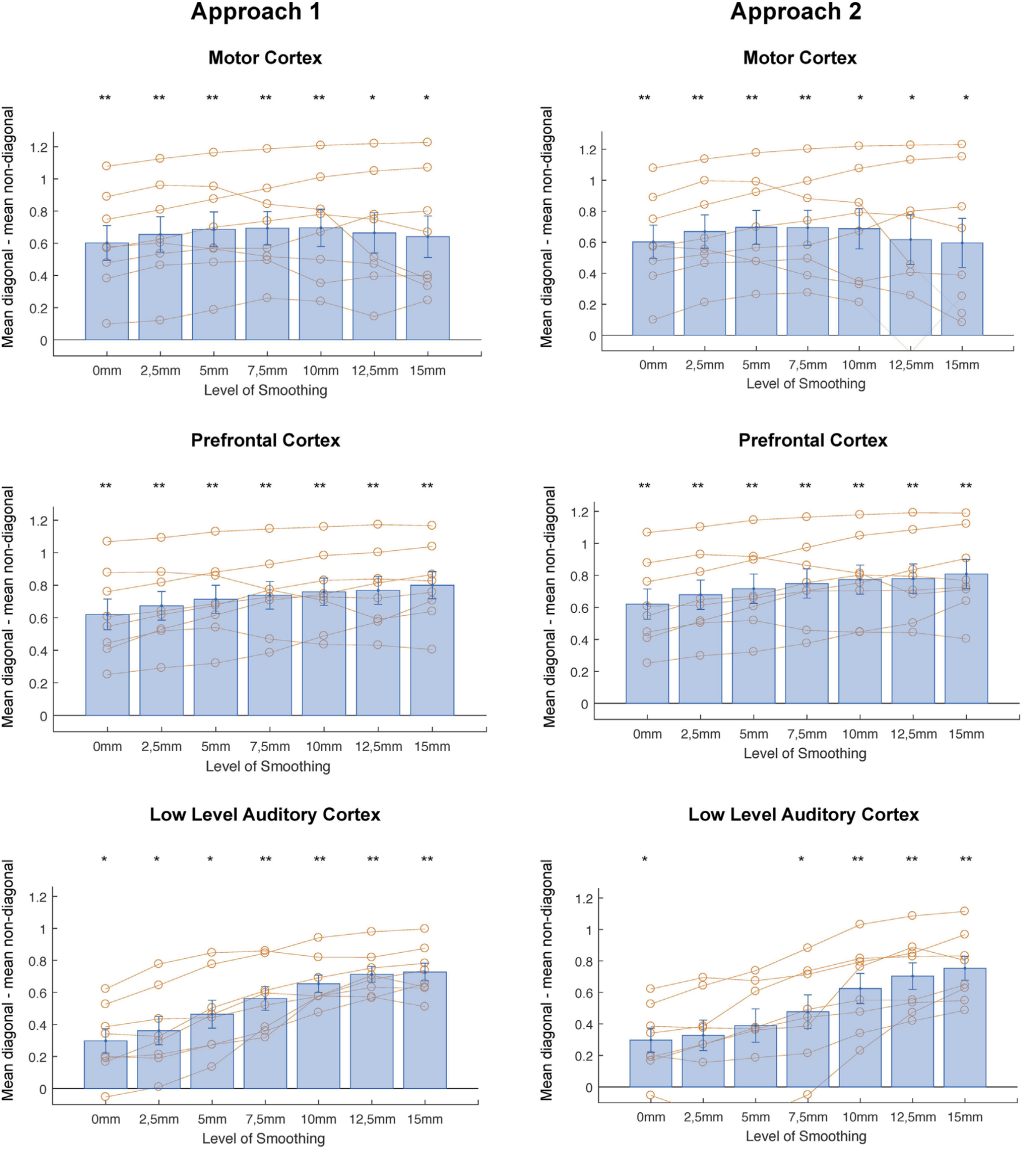

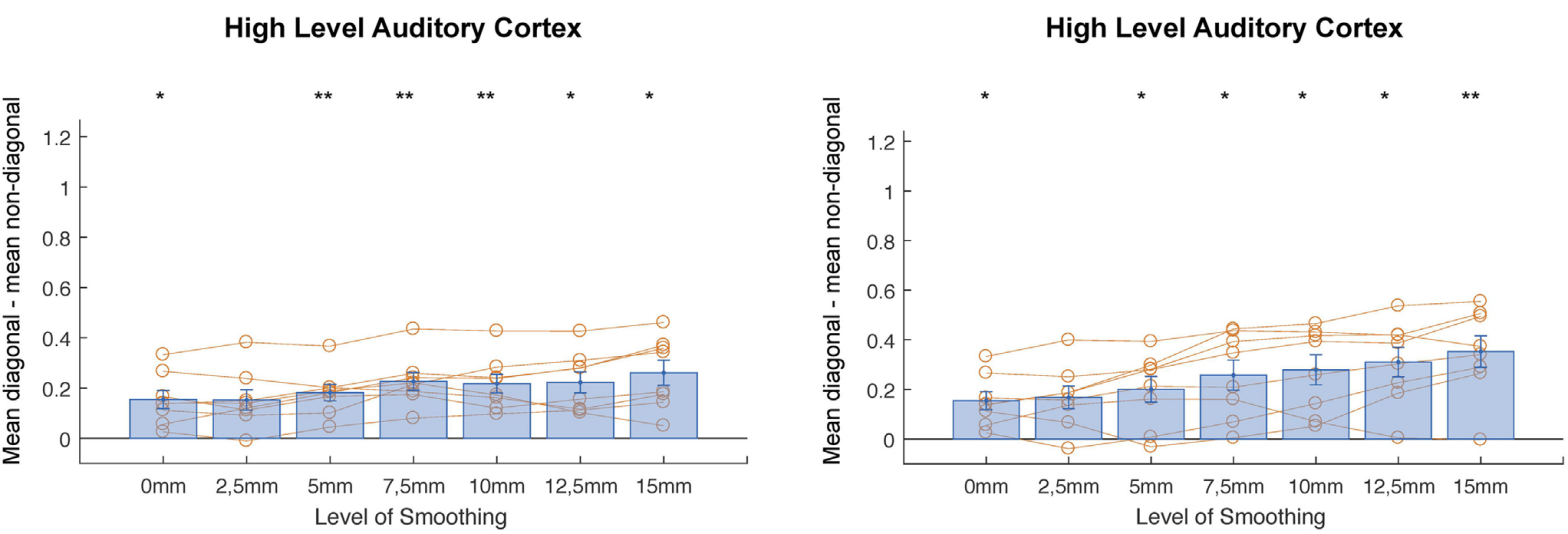
**Differences between mean diagonal and mean non-diagonal for different levels of smoothing in the four regions of interest and both approaches (Left: smoothing as part of preprocessing, Right: smoothing after masking of beta-images)**. *p < 0.0071 (Bonferroni corrected: 0.05/7), **p < 0.001. Error bars represent standard errors of the mean. Orange dots show data points for individual subjects.

In correlational MVPA, we expect correlations to be higher when we correlate patterns of the same condition than when correlating patterns of different condition. Said otherwise, we expect the diagonal cells of the correlation matrices to contain higher values compared to the non-diagonal cells (Figure 2). To study this, we first compared the mean correlations on the diagonal to the mean correlations on the non-diagonal and found that this difference was significantly higher than zero for every level of smoothing in every region, except 2.5 mm FWHM in high-level auditory cortex (Figure 3, left column) (*t*-test across subjects with a Bonferroni corrected alpha-level of 0.0071 (0.05/7) per region; motor cortex: *t*(7) > 4.9726, *p* < 0.0016 for all levels of smoothing; prefrontal cortex: *t*(7) > 6.5413, *p* < 0.0003 for all levels of smoothing; low-level auditory cortex: *t*(7) > 3.8823, *p* < 0.006 for all levels of smoothing; high-level auditory cortex: *t*(7) > 3.7435, *p* < 0.0072 for all levels of smoothing).

In addition, we examined whether the comparison between diagonal and non-diagonal was affected by smoothing within each region. When inspecting the graphs, we can see a positive trend: the difference between diagonal and non-diagonal seems to increase with a higher level of smoothing, which in a few regions increased up to the highest level of smoothing. Statistically speaking, we only found a main effect of smoothing in low-level auditory cortex, not in the three other areas [one-way ANOVA per region; motor cortex: *F*_(6,49)_ = 0.09, *p* = 0.9974; prefrontal cortex: *F*_(6,49)_ = 0.5, *p* = 0.805; low-level auditory cortex: *F*_(6,49)_ = 5.81, *p* = 0.0001; high-level auditory cortex: *F*_(6,49)_ = 1.05, *p* = 0.4078].

In a last step, we performed a two-way ANOVA with factors smoothing and brain region to test the presence of an interaction between amount of smoothing and brain region. We found a main effect of brain region [*F*_(3,196)_ = 55.86, *p* < 0.0001], a main effect of smoothing [*F*_(7,196)_ = 3, *p* = 0.0079], but no interaction [*F*_(18,196)_ = 0.18, *p* < 0.981].

#### Smoothing and Spatial Scales

**Figure 4:**
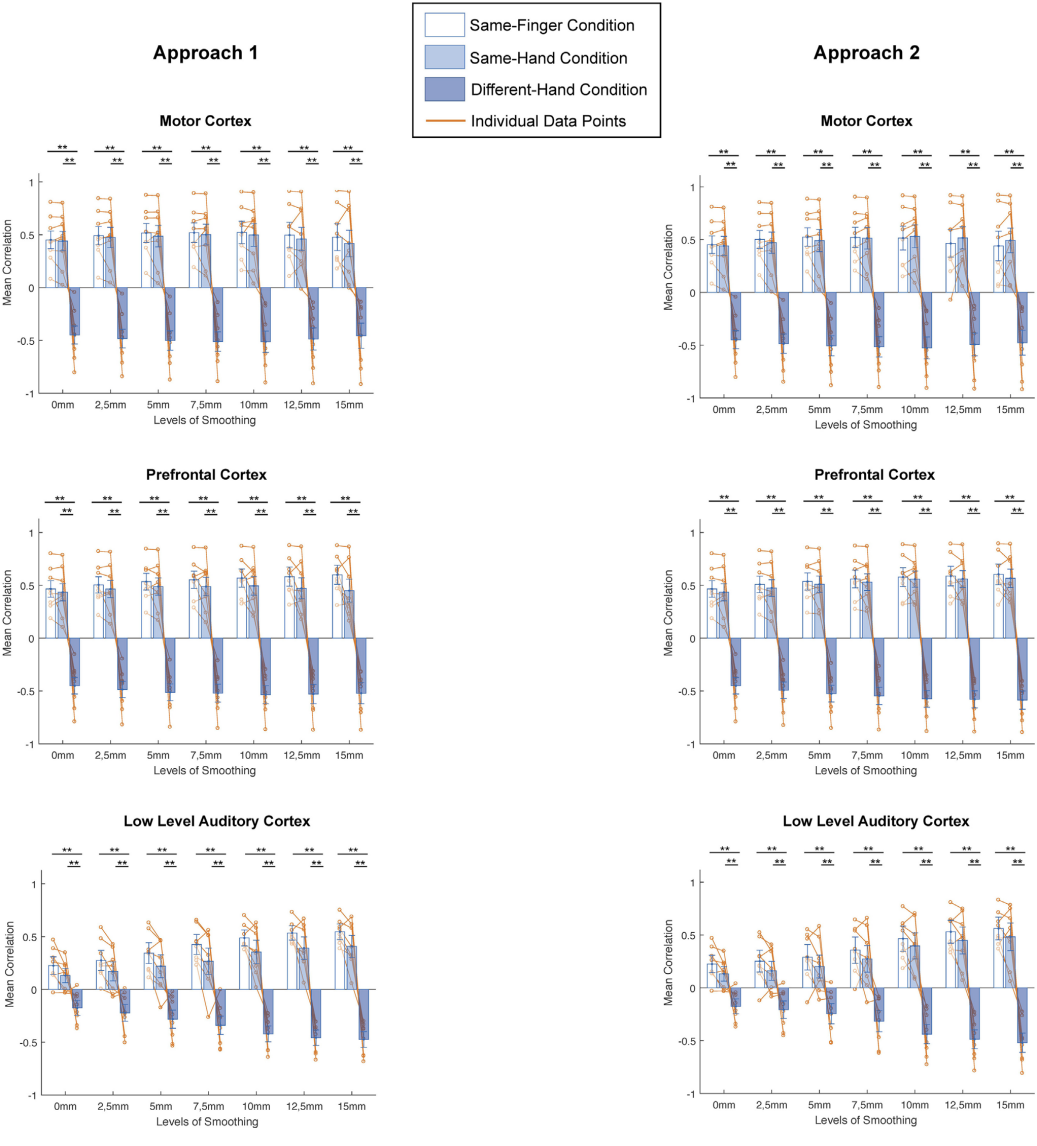

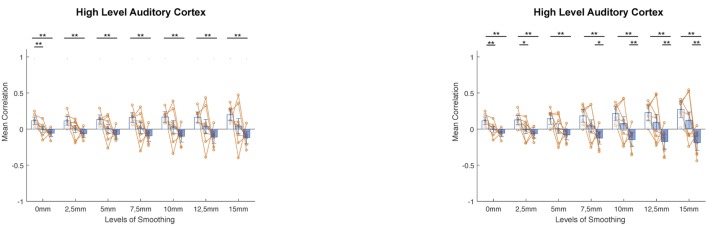
**Comparisons between different conditions for distinct smoothing levels in all studied regions of interest and both approaches (left: smoothing as part of preprocessing, right: smoothing after masking of beta-images)**. *p < 0.0024 (Bonferroni corrected: 0.05/21), **p < 0.001. Error bars represent standard errors of the mean, Orange dots represent data points of individual subjects.

Next, does smoothing affect MVPA results in a different way when looking at different spatial scales? We computed the mean correlation for every condition in all regions and investigated the difference in correlation between conditions for every level of smoothing and for every subject (Figure 4, left column). On these data, we applied a two-way ANOVA per region and found a main effect of condition in each region [motor cortex: *F*_(2,147)_ = 522.34, *p* < 0.0001; prefrontal cortex: *F*_(2,147)_ = 1002.71, *p* < 0.0001; low-level auditory cortex: *F*_(2,147)_ = 316.48, *p* < 0.0001; high-level auditory cortex: *F*_(2,147)_ = 68.95, *p* < 0.0001], a main effect of smoothing in motor and prefrontal cortex [motor cortex: *F*_(6,147)_ = 3.06, *p* = 0.0076; prefrontal cortex: *F*_(6,147)_ = 3.31, *p* = 0.0044] but not low-level and high-level auditory cortex [low-level auditory cortex: *F*_(6,147)_ = 1.62, *p* = 0.1452; high-level auditory cortex: *F*_(6,147)_ = 0.85, *p* = 0.5331] and an interaction effect in each region [motor cortex: *F*_(12,147)_ = 12.7, *p* < 0.0001; prefrontal cortex: *F*_(12,147)_ = 13.98, *p* < 0.0001; low-level auditory cortex: *F*_(12,147)_ = 7.27, *p* < 0.0001; high-level auditory cortex: *F*_(12,147)_ = 08.34, *p* < 0.0001].

In addition, we examined the results across conditions per region, using a Bonferroni corrected *p*-value of 0.0024 (0.05/21). There were clear similarities between the patterns in motor cortex, prefrontal cortex, and low-level auditory cortex. Namely, we found no significant differences between the same-finger and the same-hand conditions [*t*(62) < 2.2751, *p* > 0.0264 in all cases], but a significant difference between same-finger and different-hand conditions [*t*(94) > 8.7428, *p* < 0.0001 in all cases] and the same-hand and different-hand conditions [*t*(94) > 7.1889, *p* < 0.0001 in all cases]. On the other hand, in high-level auditory cortex, we only found a consistent significant difference between the same-finger and different-hand conditions [*t*(94) >, *p* < 0.0001] and between the same-finger and same-hand without smoothing conditions [*t*(62) = 3.6373, *p* < 0.0001]. However, we did not find a significant difference between the same-hand and different-hand conditions [*t*(94) < 2.7948, *p* > 0.0063 in all cases].

### Approach 2: Smoothing after Masking ROIs

#### Effect of Smoothing

Identically to the results for the first approach, we compared the mean correlations on the diagonal to the mean correlations on the non-diagonal and found that this difference was significantly higher than zero for every level of smoothing in every region, except 2, 5, and 5 mm FWHM in low-level auditory cortex and 2.5 mm FWHM in high-level auditory cortex (Figure 3, right column) [*t*-test across subjects with a Bonferroni corrected alpha-level of 0.0071 (0.05/7) per region; motor cortex: *t*(7) > 3.7547, *p* < 0.0071 for all levels of smoothing; prefrontal cortex: *t*(7) > 6.5413, *p* < 0.0003 for all levels of smoothing; low-level auditory cortex: *t*(7) > 3.3961, *p* < 0.0115 for all levels of smoothing; high-level auditory cortex: *t*(7) > 3.7071, *p* < 0.0076 for all levels of smoothing].

In addition, we again examined whether the comparison between diagonal and non-diagonal was affected by smoothing within each region. When inspecting the graphs, we can, as before, see a positive trend: the difference between diagonal and non-diagonal seems to increase with a higher level of smoothing. In contrast, as for the first approach, we only found a main effect of smoothing in low-level auditory cortex, not in the three other areas [one-way ANOVA per region; motor cortex: *F*_(6,49)_ = 0.12, *p* = 0.9931; prefrontal cortex: *F*_(6,49)_ = 0.51, *p* = 0.7997; low-level auditory cortex: *F*_(6,49)_ = 3.96, *p* = 0.0026; high-level auditory cortex: *F*_(6,49)_ = 1.86, *p* = 0.1076].

In a last step, we again performed a two-way ANOVA to test the presence of an interaction between amount of smoothing and brain region. We found a main effect of brain region [*F*_(3,196)_ = 34.96, *p* < 0.0001], a main effect of smoothing [*F*_(7,196)_ = 2.67, *p* = 0.0163], but no interaction [*F*_(18,196)_ = 0.78, *p* < 0.7173].

#### Smoothing and Spatial Scales

A next question was whether smoothing affects MVPA results in a different way when looking at different spatial scales. We computed the mean correlation for every condition in all regions and investigated the difference in correlation between conditions for every level of smoothing and for every subject (Figure 4, right column). On these data, we applied a two-way ANOVA per region and found a main effect of condition in each region [motor cortex: *F*_(2,147)_ = 501.63, *p* < 0.0001; prefrontal cortex: *F*_(2,147)_ = 1261.64, *p* < 0.0001; low-level auditory cortex: *F*_(2,147)_ = 213.49, *p* < 0.0001; high-level auditory cortex: *F*_(2,147)_ = 79.25, *p* < 0.0001], a main effect of smoothing in motor and prefrontal cortex [motor cortex: *F*_(6,147)_ = 3.04, *p* = 0.0078; prefrontal cortex: *F*_(6,147)_ = 4.17, *p* = 0.0007] but not low-level and high-level auditory cortex [low-level auditory cortex: *F*_(6,147)_ = 1.25, *p* = 0.2852; high-level auditory cortex: *F*_(6,147)_ = 1.73, *p* = 0.1184], and an interaction effect in each region [motor cortex: *F*_(12,147)_ = 13.2, *p* < 0.0001; prefrontal cortex: *F*_(12,147)_ = 16.48, *p* < 0.0001; low-level auditory cortex: *F*_(12,147)_ = 5.85, *p* < 0.0001; high-level auditory cortex: *F*_(12,147)_ = 8.73, *p* < 0.0001].

Furthermore, we examined the results across conditions per region, using a Bonferroni corrected *p*-value of 0.0024 (0.05/21). Even more so than for the first approach, there were clear similarities between the patterns in motor cortex, prefrontal cortex, and low-level auditory cortex. Namely, we found no significant differences between the same-finger and the same-hand conditions [*t*(62) < 1.7469, *p* > 0.0856 in all cases], but a significant difference between same-finger and different-hand conditions [*t*(94) > 8.3309, *p* < 0.0001 in all cases], and the same-hand and different-hand conditions [*t*(94) > 7.0811, *p* < 0.0001 in all cases]. In high-level auditory cortex, we also found a consistent significant difference between the same-finger and different-hand conditions [*t*(94) > 5.0441, *p* < 0.0001]. However, the difference between the same-hand and different-hand conditions was only significant in the four biggest levels of smoothing [*t*(94) > 3.2512, *p* < 0.0016 in all significant cases], and the difference between the same-finger and same-hand conditions was only significant for results without smoothing and with smoothing of 2.5 mm FWHM [*t*(62) = 3.2404, *p* < 0.0019 for the two significant cases].

## Discussion

To summarize, smoothing does not seem to degrade the results of correlational MVPA in this combined motor/auditory paradigm. More specifically, the difference between diagonal and non-diagonal was significant in almost all smoothing levels in almost all studied regions, in both approaches. This means we can reliably distinguish activity from conditions differing in motor and auditory dimensions based on neural data with almost all smoothing levels in all studied regions in both approaches. Averaged across all regions, there was an overall positive effect of smoothing. In motor cortex, our results show a different pattern than the other three regions, namely, a decrease of information for the higher levels of smoothing (i.e., 12.5 and 15 mm FWHM). On the other hand, in the other three regions and most obviously low-level auditory cortex smoothing even with the largest kernel will not hurt MVPA results and if anything it seems to have a slight positive effect.

The effect of smoothing can be explained using various factors. Chaimow et al. (5) explained several mechanisms that possibly account for hyperacuity. The fact that results of (correlational) MVPA are fairly robust to smoothing, is at face value not hyperacuity (9). Sengupta et al. (25) speculated about the Nyquist criterion: with a particular sampling frequency, we can only measure frequencies up to half the sampling frequency. In this case, it would mean that with a voxel size of 2.5 mm, we could measure frequencies up to 5 mm. In all regions, the signal clearly increased up to 5 mm smoothing (and further), so it seems possible this criterion plays a role. We cannot, however, exclude other explanations such as draining veins or local, random variations in the brain’s functional organization. Furthermore, besides the effect of smoothing, clear differences between the regions of interest can be noticed, at least in the overall trend in the figures, because the regional differences did not reach significance with our small sample size. An obvious explanation for regional differences could be the size of the region, operationalized by the number of voxels. However, only prefrontal cortex is a lot bigger than the other three, hence it does not seem a good explanation. Another possibility is that regions differ in the scale of their functional organization. The larger this scale is, the more benefit we can expect from higher levels of smoothing.

We used two approaches to analyze the data. For the first approach, we followed the fMRI data analysis pipeline that is often used, in which smoothing is part of the preprocessing. It is important to include this approach in order to use the current findings to make predictions about potential effects in other studies that might most frequently use this smoothing approach. However, there is a major drawback of this type of smoothing, given that noise from voxels outside an ROI (which can even be in white matter) could contaminate the signal from inside the ROI (13). This effect gets stronger the bigger the amount of smoothing. In the second approach, the data of voxels outside the ROI are excluded, thus no longer influencing the outcome of the smoothing, which only involves the signal from voxels inside the ROI. When comparing these two approaches, we did not notice major differences, meaning the large trends remain the same for the two approaches. Nevertheless, the alteration in the way we analyzed the data did cause some small changes in the results (e.g., results that are not significant for the second approach that were for the first one when looking at the effect of smoothing) and more pronounced changes in individual patterns for each participants. Another possible way to approach the analysis of these data would be to use spatial band pass filtering, which has been shown to affect MVPA results as well (13, 25). A full exploration of all possible filtering approaches is beyond the scope of the current study in which we focus upon the most commonly used approach of spatial smoothing. Nevertheless, bandpass filtering is a very fruitful approach to obtain additional insight as to why MVPA results are influenced by filtering of the data.

Overall, the findings suggest that a moderate level of smoothing does not hurt MVPA findings and, if anything, can provide a moderate improvement. The results seem to be largely in line with the findings of Op de Beeck (9). He found that the effect size of correlational MVPA was higher when using various levels of smoothing compared with no smoothing in primary visual and lateral occipital cortex. A parallel decoding MVPA approach showed in addition that relatively large amounts of spatial smoothing do not hurt results of decoding MVPA. Note, however, that at least in some studies, larger amounts of smoothing have been shown to induce a decrease in decoding accuracy after smoothing (29, 30). In addition, Misaki et al. (30) obtained a fairly similar finding with regard to the pattern of individual subjects when performing several levels of smoothing and evaluating the decoding accuracies: each subject’s pattern over different levels of smoothing was distinct from the patterns of other subjects. Note that in our study, there are also individual differences, but they could be simply due to individual noise.

When comparing different conditions, we found the same pattern reoccurring for all levels of smoothing in motor, prefrontal, and low-level auditory cortices. Namely, we can distinguish between neural activity coming from different hands, but not from the same hand. Even without smoothing, we found no significant difference between the same-finger and same-hand conditions. This seems to contradict the idea of hyperacuity, i.e., it is possible to pick up functional organization smaller than a voxel size using MVPA. Note that in the current study, we might simply lack the ability to pick up signals of the supposedly smallest spatial scale. Importantly, the pattern was somewhat different in the high-level auditory cortex. Only in high-level auditory cortex, we can distinguish between neural activities from same-finger and same-hand conditions for some smoothing levels, mostly for the second approach. Of course, the selectivity in auditory cortex reflects the auditory cues that are quite different in these conditions.

The question remains; how can researchers choose the optimal level of smoothing based on careful examination? Importantly, recommendations will transcend our data, and generalization is difficult because every study uses different approaches and techniques, and even results in distinct regions differ when performing the exact same analyses. For example, when looking at motor cortex, we see that smoothing with a 5, 7.5, or 10 mm kernel would probably be best in both approaches, as both the difference between mean diagonal and mean non-diagonal and the difference between the same- versus different-hand conditions are optimized. The optimal level would therefore be one of those. Which one exactly should not matter too much, since such small difference should not affect the results when findings are strong and consistent. For the other regions in this article, we might have chosen different optimal levels of smoothing (as they show a different trend). In general, our results show that smoothing is advantageous when compared to no smoothing in most regions, which is the most important observation to make and for readers to remember. Examining the effect of smoothing is vital when researchers suspect it to have an effect on their results based on, for example, the organization of the region they will study.

In conclusion, we observed minor effects of smoothing in this paradigm. Spatial smoothing is only one of many parameters chosen by researchers during their analyses, and they should be aware of the effect of seemingly arbitrary choices on the outcome of their study. In the context of robustness of results, it has been argued that conclusions should remain the same regardless of the arbitrary choices made during data analysis (31). With the small sample size in the current study, there were several examples where the exact choice of smoothing level affected whether results are found to be significant. Although it might seem helpful to state a general rule on the best smoothing level to use, this is a difficult—if not impossible—recommendation to make. The effect of smoothing is not necessarily the same for different brain regions. Hence, we emphasize the importance of thinking about this choice. As an interesting example, Gardumi et al. (32) studied the effect of smoothing on their dataset and decided to use the optimal smoothing level only after careful examination.

## Ethics Statement

This study was carried out in accordance with the recommendations of the medical ethics committee of the KU Leuven with written informed consent from all subjects. All subjects gave written informed consent in accordance with the Declaration of Helsinki. The study served as a control experiment in a more extensive study, of which the protocol was approved by the medical ethics committee of the KU Leuven.

## Author Contributions

MH: recruitment of participants, data collection, including programming the script (which was an adaptation of the script of FP), operating the fMRI scanner, paperwork, contacting subjects, data analysis, adapting existing code (adapted script of FP) for preprocessing, univariate and multivariate analyses, and writing the first draft and adapting next versions. ND: data collection, including operating fMRI scanner and paperwork, practical assistance during all steps, and proofreading drafts. FP: paradigm development, provided scripts and support with adaptation, support during analyses and feedback during all stages of the experiment, and proofreading drafts. HB: paradigm development. Feedback and support during all steps, in particular data collection, data analysis, and interpretation of data. Writing and support during adaptation of drafts.

## Conflict of Interest Statement

The authors declare that the research was conducted in the absence of any commercial or financial relationships that could be construed as a potential conflict of interest.
